# Impact of Age and Heart Rate on Strain-Derived Myocardial Work in a Population of Healthy Subjects

**DOI:** 10.3390/diagnostics12071697

**Published:** 2022-07-12

**Authors:** Ciro Santoro, Federica Ilardi, Roberta Esposito, Giulia Elena Mandoli, Mario Enrico Canonico, Federica Buongiorno, Grazia Canciello, Maria Prastaro, Maria-Angela Losi, Giovanni Esposito

**Affiliations:** 1 Department of Advanced Biomedical Science, Federico II University Hospital, 80131 Naples, Italy; fedeilardi@gmail.com (F.I.); mecanonico@gmail.com (M.E.C.); federicabuongiorno94@gmail.com (F.B.); grazia.canciello@hotmail.it (G.C.); prastaro@unina.it (M.P.); losi@unina.it (M.-A.L.); espogiov@unina.it (G.E.); 2 Department of Clinical Medicine and Surgery, Federico II University Hospital, 80131 Naples, Italy; roberta.esposito6@gmail.com; 3 Department of Medical Biotechnologies, Division of Cardiology, University of Siena, 53100 Siena, Italy; giulia_elena@hotmail.it

**Keywords:** global longitudinal strain, myocardial work, heart rate, age, myocardial mechanics

## Abstract

Background: The influence of age and gender on strain-imaging-derived myocardial work (MW) was recently investigated in healthy subjects. No information is available on the impact of heart rate (HR) on MW. Methods: 177 healthy subjects (47% men, mean age 42 years) underwent an echo-Doppler exam, including quantification of global longitudinal strain (GLS). Cuff blood pressure was used as a surrogate of left ventricular peak pressure to estimate global work index (GWI), global constructive work (GCW), global wasted work (GWW) and global work efficiency (GWE). Statistical analyses were performed according to age and HR tertiles. Results: GWW was higher in the third HR tertile, i.e., ≥74 bpm (74.7 ± 33.6 mmHg %) than in the first HR tertile (<66 bpm) (61.0 ± 32.5 mmHg %) (*p* < 0.02). In the pooled population, by adjusting for systolic blood pressure, GLS, E/e’ ratio and left atrial volume index, age was independently associated with GCW (β = 0.748) and GWI (β = 0.685) (both *p* < 0.0001) and HR with GWW (β = 0.212, *p* = 0.006) and GWE (β = −0.204, *p* = 0.007). Conclusions: In healthy subjects age shows a mild influence on GCW. HR exerts an independent negative impact on GWW and GWE: the higher HR the greater wasted work and lower myocardial efficiency.

## 1. Introduction

The progressive advancement of cardiac ultrasound has led to large clinical use of two-dimensional (2D) speckle tracking echocardiography (STE). Global longitudinal strain (GLS), in particular, allows quantifying the degree of left ventricular (LV) longitudinal dysfunction beyond ejection fraction (EF). The utilization of GLS is promoted by the echocardiographic scientific societies [1,2], also in relation with its high feasibility and reproducibility, which makes it suitable for serial assessment over time. GLS has been recently also employed to address cardioprotective therapy in cancer patients with subclinical LV dysfunction and successfully prevent progression towards overt heart failure [3]. Additionally, in the oncologic setting GLS represent a valuable tool to detect immune checkpoint inhibitor-related myocarditis, being associated with cardiovascular events independently from LV EF [4] Besides these recognized advantages, similarly to EF, GLS presents the limitation of being largely load-dependent, which hampers a reliable evaluation of myocardial function when preload and afterload changes occur [5,6]. In particular, the afterload increase has been demonstrated to reduce GLS, inducing possible misapprehension of the true LV systolic function state [7,8].

More recently, according to the assumption that the invasively determined LV pressure–volume loop reflects stroke work [9,10,11,12], the concept of myocardial work (MW) has been transferred to STE imaging. It takes into account deformation as well as afterload, through interpretation of GLS in relation to clinically measured systolic blood pressure (BP) [13]. This methodology, originally proven in experimental dog models [13,14], has been successfully validated to quantify regional and global myocardial work and provide information regarding metabolic demand in humans [14]. Different components of non-invasive MW can be determined and include global constructive work (GCW), global wasted work (GWW) and global work efficiency (GWE). Clinical studies have demonstrated the practical usefulness of strain-derived MW in several pathological conditions, such as desynchronized ventricles, non ST-segment elevation acute coronary syndromes, arterial hypertension and dilated cardiomyopathy [15,16,17]; MW also predicts results of percutaneous edge-to-edge mitral valve repair [18] and both therapy response and mortality after cardiac resynchronization therapy [19,20,21].

Determinants of MW and its components in healthy subjects have already been investigated by the multicenter EACVI NORRE Study. It provided evidence on the absence of a strong influence of age and sex on MW and also highlighted the association of both global work index (GWI) and GCW with systolic BP [22]. The present study extends the investigation to the possible impact of heart rate (HR), which is known to negatively impact cardiovascular outcomes [23], on non-invasive MW in a single-center population of healthy subjects.

## 2. Materials and Methods

### 2.1. Study Population

The study population included 177 consecutive healthy adult subjects, prospectively recruited on a voluntary basis from January to December 2018. The informed consent of each participant was collected. No subject had cardiovascular risk factors, such as arterial hypertension (BP > 140/90 mmHg), dyslipidemia (total cholesterol > 190 mg/dL and/or triglycerides > 150 mg/dL), diabetes mellitus (fasting glycaemia > 100 mg/dL), obesity (body mass index > 29.9 kg/m^2^), and smoke habit. Sinus tachycardia (HR > 100 bpm) and age < 18 years were the main exclusion criteria. Subjects with a history of coronary artery disease and previous acute myocardial infarction, stroke, transient ischemic events, more than mild valvular heart diseases, congestive heart failure, primary cardiomyopathies, congenital heart diseases, systemic diseases, active or previous history of cancer, pharmacological therapies, any kind of resting electrocardiographic abnormalities and echocardiograms of poor quality, were also excluded.

### 2.2. Standard Echocardiographic Procedures

A standard Doppler echocardiographic exam was performed according to standardized procedures of our laboratory [24,25] by Vivid E95 (GE Healthcare, Horten, Norway) machine, by using a 2.5 MHz phased array transducer. At the beginning of the exam, a physician blinded to the examination estimated cuff brachial BP (the mean of three measurements) and HR from an ECG trace obtained during apical long-axis view recording. Echocardiographic acquisitions were performed according to the ASE/EACVI Chamber quantification recommendations and the EACVI standardization of the echocardiographic report [2,26]. Standard echo parameters included LV mass and relative wall thickness, 2D-derived LV volumes and EF (Simpson biplane), left atrial volume index and Doppler measurements of LV diastolic function (transmitral E/A ratio and E velocity deceleration time, pulsed tissue Doppler-derived e’ velocity of lateral and septal mitral annulus, and E/e’ average ratio) [2]. STE-derived longitudinal strain acquisition and post-processing were performed in apical long-axis, 4-chamber and 2-chamber views by Automated Function Imaging software according to standardized procedures of our laboratory [24,25]. Segmental peak systolic strain was measured for each of the six LV walls (antero-septal, infero-septal, inferior, inferolateral, lateral and anterior wall), obtaining a 16-segments bull’s eye plot. GLS was calculated by averaging all values of regional peak systolic longitudinal strain.

### 2.3. Two-Dimension Speckle Tracking Derived MW

**Figure 1:**
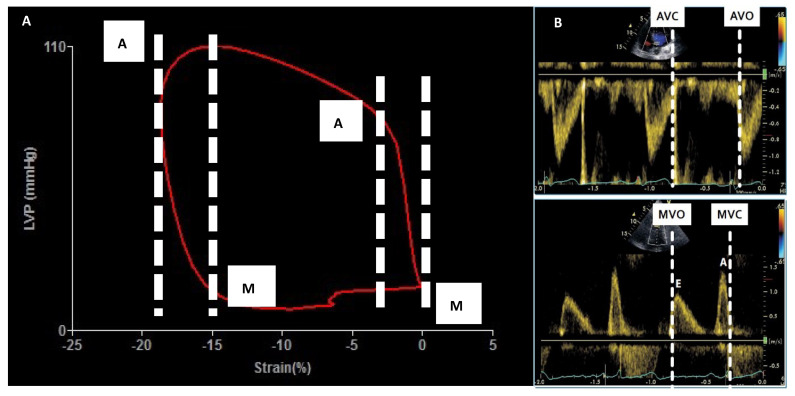
Methodology for measuring strain-derived myocardial work. In the left side the strain pressure loop: the area inside the loop corresponds to global work index (panel **A**). In the right panel Doppler-derived measurement needed to mark systolic and diastolic time intervals (panel **B**). AVC = Aortic valve closure, AVO = Aortic valve opening, LVP = Left ventricular pressure, MVC = Mitral valve closure, MVO = Mitral valve opening.

The quantitative analysis of MW was performed using a commercially available, vendor-specific software package (Echopac V. 2.03, GE). By using this software and according to standardized procedures [13], cuff systolic BP is assumed to be equal to LV peak systolic pressure and combined with acquired GLS [23,24], in order to construct a noninvasive pressure–strain loop (Figure 1A).

The patient-specific LV pressure curve is built by adjusting the standard LV pressure curve to the duration of isovolumic and ejection phases defined by the timing of valvular events (marked by previously recorded Doppler imaging) (Figure 1B). Strain and pressure data are synchronized using the ECG-derived R-wave onset, providing pressure–strain loop curves whose areas express the global work index (GWI), which is evaluated from mitral valve closure to mitral valve opening (Figure 1A). Among the MW components, constructive work (mmHg %) of myocardial segments is defined as the sum of effective work produced during segmental shortening in systole and during lengthening of isovolumic relaxation time being representative of the total amount of energy that actively contributes to the systolic work, expressed by the formula:

GCW = systolic shortening + lengthening during IVRT

On the other hand, wasted work (mmHg %) computed as the sum of the energy spent for the lengthening in systole and shortening in isovolumic relaxation, correspond to energy loss spent during ineffective work expressed by the formula:

GWW = systolic lengthening + shortening during IVRT

The ratio between the constructive work and the sum of constructive and wasted work represents myocardial work efficiency (%):

GWE = Constructive MW/(Constructive MW + Wasted MW)

Global values are obtained as averages from all segmental values of MW parameters, including GWI, GCW, GWW, and GWE.

### 2.4. Statistical Analyses

All statistical analyses were performed by the SPSS software, release 12 (SPSS Inc., Chicago, IL, USA, version 12). The normal distribution of continuous variables was tested by the Kolmogorov–Smirnov test. All data were expressed as mean ± standard deviation (SD). Descriptive statistics were obtained by one factor ANOVA and χ^2^ distribution with computation of exact p value by the Monte Carlo method. Participants were divided into tertiles of age and HR, and analyses were performed by using post-hoc inter-group Bonferroni test. Correlation between continuous variables was tested by Pearson’s correlation coefficient. Multivariable linear regression analyses were performed to examine the independent correlates between MW components and both demographic and echocardiographic variables. Collinearity was considered acceptable and regression model stable for variance inflation factor less than 3. Reproducibility tests of chosen variables (MW work components) were determined by measuring average intra-class correlation coefficients (ICC) and their 95% confidence intervals (CI). Average ICC > 0.80 were considered to be optimal. The null hypothesis was rejected at two-tailed *p* < 0.05.

## 3. Results

Table 1 summarizes the demographics and main echo-Doppler parameters including GLS and MW components. Mean age was 42 ± 16 years and HR 71 ± 10 bpm. Twenty-nine subjects were older than 60 years and 31 had HR ≥ 80 bpm (data not in Table). GCW was 2566 ± 348 mmHg and GWE 96.7 ± 1.5 mmHg. Notably, no significant difference was found between women and men relating to GWI (2288 ± 342 vs. 2275 ± 358 mmHg %; *p* = 0.10), GCW (2584 ± 349 vs. 2548 ± 349 mmHg %; *p* = 0.82), GWW (68 ± 34 vs. 69 ± 31 mmHg %; *p* = 0.82) and GWE (96.8 ± 1.4 vs. 96.6 ± 1.6 mmHg %; *p* = 0.61). The univariate analyses showed the following significant relations: age with GCW (r = 0.20, *p* = 0.009), systolic BP with GWI (r = 0.65, *p* < 0.0001), GCW (r = 0.72, *p* < 0.0001) and GWW (r = 0.15, *p* < 0.05), diastolic BP with GWI (r = 0.33) and GCW (r = 0.34) (both *p* < 0.0001), HR with GWW (r = 0.19, *p* < 0.01) and GWE (r = −0.16, *p* = 0.03). GWI was also related with E/e’ ratio (r = 0.22, *p* = 0.004) and left atrial volume index (r = 0.22, *p* = 0.003), GCW with EF (r = 0.15, *p* < 0.05), E/e’ ratio (r = 0.21, *p* < 0.005) and left atrial volume index (r = 0.21, *p* < 0.005). (Supplementary Table S1).

**Figure 2:**
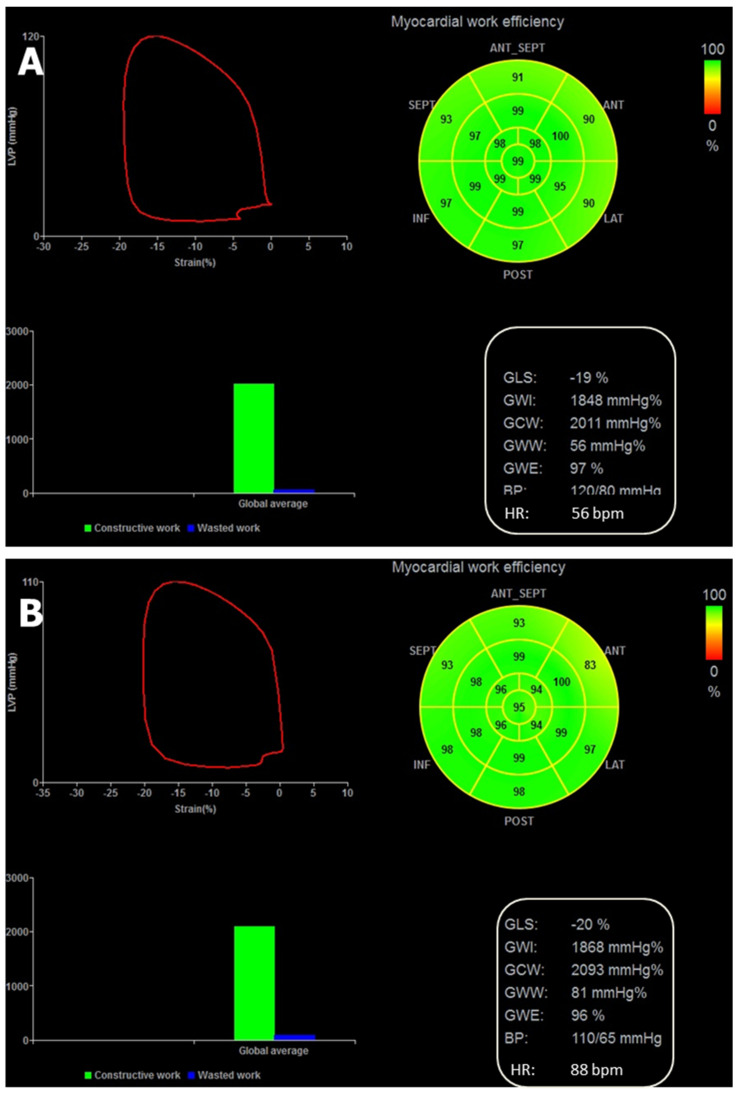
The strain-derived MW analysis of two healthy subjects with similar age, the first with sinus bradycardia and the second one with HR = 88 bpm: GWW is substantially higher in the subjects with faster HR. Strain-Pressure (LVP) loop (upper left panel of each figure), regional myocardial efficiency bull’s eye (upper right panel), diagram of average constructive and wasted work percentages (lower left panel) and list of global work components (lower right panel) in a 57-year-old healthy female subject with sinus bradycardia (HR = 56 bpm) (**A**) and in a 58-year-old healthy male subject with HR = 88 bpm (**B**). In presence of marginal changes of the global constructive work, the percentage of global wasted work is relatively low in the patient with sinus bradycardia (**A**) whereas it appears to be substantially increased in the patient with HR = 88 bpm (**B**). GCW = Global constructive work, GWE = Global work efficiency, GWW = Global wasted work, GWI = Global work index.

Table 2 reports the analysis of MW components according to age tertiles (<32, between 32 and 49, ≥49 years) and HR (<66, between 66 and 74, ≥74 bpm). According to age subdivision, subjects ≥49 years presented higher GCW than subjects <32 years (*p* < 0.01), whereas GWW and GWE did not differ among the three tertiles. According to HR tertiles, subjects with HR ≥ 74 bpm had higher GWW than subjects with HR < 66 bpm (*p* < 0.02). The impact of age and HR on MW parameters are represented in the explicatory clinical case comparison in Figure 2. Supplementary Table S2 depicts distribution of demographic and echocardiographic data according to age tertiles. Correlation was found between HR and age (r = −0.19; *p* > 0.01).

Table 3 shows the results of separate multilinear regression analyses considering MW components as dependent variables. After adjusting for SBP, E/e’ ratio and left atrial volume index and GLS, age remained independently associated only with GWI, whereas HR was independently associated with GWI, GWW and GWE. Systolic BP was independently associated with GWI and GCW but did not enter the models of GWW and GWE.

By testing the MW components reproducibility in a subgroup of 30 healthy subjects, the Intra-observer ICC were the following: GLS = 0.97 (95% CI = 0.92–0.99), GWI = 0.95 (95% CI = 0.86–0.98), GCW = 0.99 (95% CI = 0.99–1.00), GWW = 0.97 (95% CI = 0.89–0.99), GWE = 0.95 (95% CI = 0.84–0.98) (all *p* < 0.0001). The interobserver ICC were: GLS = 0.92 (95% CI = 0.73–0.97), GWI = 0.93 (95% CI = 0.78–0.98), GCW = 0.95 (95% CI = 0.84–0.98), GWW = 0.94 (95% CI = 0.82–0.98), GWE = 0.89 (95% CI = 0.68–0.97) (all *p* < 0.0001).

## 4. Discussion

No method exists in order to non-invasively record myocardial force–dimension loops, because force calculation needs measurements not simultaneously and continuously obtainable during the cardiac cycle. The strain-derived loop replaces force with BP and allows determining LV pressure–strain area as an index of MW [12,13,14,15]. Despite a still-small body of evidence, the evaluation of MW could provide potential additional value to stratify prognosis and address management of patients across a broad range of physiologic and pathologic cardiovascular conditions [25,26,27,28]. In order to optimize the use of this method in the clinical setting it is mandatory to know the physiological determinants of pressure–strain relation in healthy subjects. The NORRE Study explored this issue for the first time, by testing the possible impact of age, gender and systolic BP on MW components [21]. The present study extends the investigation to the possible effect of HR and some other main variables, obtainable by standard echocardiography, on MW components. Our findings demonstrate that in a single-center population of healthy subjects (I) systolic BP strongly influences GWI and GCW but does not present an independent impact on GWW and GWE, (II) age exerts a marginal effect on MW components, with an independent inverse association only with GWI and a significant increase of GCW in subjects ≥49 years, (II) HR ≥ 74 bpm induces a significant GWW increase and, in general, higher HR is independently associated with GWW increase and GWE reduction. 

The association of systolic BP with GWI and GCW was already highlighted by the NORRE Study, which also showed a lack of age dependency on MW components [21]. The present study confirmed these data. The impact of systolic BP on GWI and GCW is not unexpected, since systolic BP represents the surrogate of invasive peak LV pressure, which allows building strain derived pressure loop. The effect of BP on GCW was clearly observed in hypertensive patients [17].

Our data also confirmed only a mild effect of age on MW in healthy subjects. In the multivariable models, age was marginally but significantly related to GWI, but its association with GCW, GWW and GWE was not independent on clinical and echo confounders. However, by dividing the population according to age tertiles, subjects ≥49 years presented higher GCW than the youngest subjects (<32 years). This data is in line with results provided from the Characteristics and Course of Heart Failure STAges A/B and Determinants of Progression (STAAB) cohort study which included 4965 participants aged 30–79 years. The authors found that GCW, GWW, and GWE were not affected by sex, BMI and age below the 45 years, whereas linear increase with GWW was evident above the age cut-off [29]

The concept that myocardial oxygen consumption increases and myocardial efficiency decreases with the heart beat increase is well known. To the best of our knowledge, the present study is the first to confirm the significant impact of HR on some MW components, i.e., GWI, GWW and GWE by applying the non-invasive, strain-imaging-derived technology. In particular, GWW was substantially increased in the third HR tertile (HR ≥ 74 bpm) and HR was positively and independently associated with GWW in the pooled population. Although the myocardial efficiency was not significantly reduced in the third HR tertile, in the multiple regression analyses HR was also inversely associated with GWE, which is indirectly derived from the ratio of GCW and GWW [17]. Of interest, the influence of faster HR on GWW increase and GWE reduction was independent on the effect of cofounders, including E/e’ ratio and left atrial volume index. The lack of independent associations of these recognized indicators of LV diastolic function [30] with MW components confirms similar results of the NORRE study [31]. We postulate that faster HR could induce a greater myocardial oxygen consumption and thus a greater amount of GWW, i.e., of the MW not contributing to LV ejection, by a possible myocyte lengthening rather than shortening during systole while a substantial proportion of myocardial segments anticipate the shortening during diastole. This will obviously lead to detrimental reflections on GWE. It is likely that the negative influence exerted by HR on MW components could be even amplified in subjects with sinus tachycardia, who were excluded by definition from the present study. The exclusion of patients with tachycardia can even explain the marginal clinical impact of HR found on myocardial efficiency of our population.

### 4.1. Study Limitations

The main limitation is intrinsic to the utilized methodology which replaces central (aortic) with cuff BP. Systolic BP varies throughout the arterial tree and cuff systolic BP is slightly overestimated in comparison with the corresponding brachial BP, with a variable inter-individual disparity [32,33]. The difference between central and cuff BP is mainly determined by the arterial stiffness and is pronounced in patients with cardiovascular diseases. Moreover, by using strain–pressure loop diastolic pressure, pressure rise and pressure fall are poorly approximated, and a marked difference between estimated and invasively measured pressures during diastole has been clearly demonstrated [6].

This is a study performed on a series of healthy individuals and the results cannot be transferred to patients with cardiac diseases. GWW is only a small fraction of global MW and the impact of its HR-related changes does not appear to be clinically relevant on myocardial efficiency of our healthy population. Accordingly, the study of relationships between HR and MW components needs to be extended to the spectrum of pathologic conditions, in particular heart failure and coronary artery disease. Moreover, the data of the present study refers to a relatively small sample size of the healthy population, with a limited number of elderly subjects, collected in a single-center enrollment and should be therefore extended to larger populations. The exclusion of subjects with sinus tachycardia, a pre-defined requirement for recruitment, can also have influenced the statistical impact of HR in the analyses performed. Accordingly, while the cumulative R2 of the multivariable models assessing GWI and GCW explained a large variance proportion (79 and 85% respectively), the cumulative R2 of GWW and GWE models were relatively small (0.18 and 0.16 respectively). It is likely that other covariates—not investigated in the present study—could exert additional influence on MW components.

### 4.2. Clinical Implication

Elevated HR at rest is a well-known indicator of cardiovascular outcome in heart failure with reduced EF [34]. One-beat HR increase augments the risk of cardiovascular death by 3% and 5-beat increase the risk of heart failure hospitalization by 16% respectively [35]. Our study indirectly supports these findings demonstrating that elevated HR could be associated with a substantial increase of ineffective work and a consequent decrease of myocardial efficiency—both unfavorable conditions for a good survival. These findings could even be amplified in heart failure patients who present a blunted force–frequency relationship at increasing HR [36].

## 5. Conclusions

The current investigation provides evidence that HR, and to a lesser extent age, should be considered when assessing strain-imaging-derived MW in the human heart. In particular, failure to adjust for HR may lead to inappropriate interpretation of MW data obtained. Further studies could be addressed to evaluate the effects of cardiac drugs producing changes of both BP and HR on MW and demonstrate possible reflections of these changes on the therapeutic management of cardiac patients.

## Figures and Tables

**Table 1:** Demographic and echo-Doppler characteristics of the study population.

Parameter	Mean ± SD	Range
Gender (M/F)	84/93	-
Age (years)	41.8 ± 15.9	18–86
BMI (Kg/m^2^)	23.3 ± 2.6	13.9–29.8
Systolic BP (mmHg)	120.5 ± 12.6	85–140
Diastolic BP (mmHg)	75.0 ± 7.8	60–90
Mean BP (mmHg)	90.2 ± 8.3	70–106.7
Heart rate (bpm)	70.6 ± 10.4	46–97
LV mass index (g/m^2^)	70.3 ± 16.5	26.9–119.7
RDWT	0.32 ± 0.05	0.19–0.46
LV EF	66.5 ± 7.0	53–79.7
E/A ratio	1.33 ± 0.40	0.70–2.45
E velocity DT	197.1 ± 33.0	107–254
E/e’ ratio	6.69 ± 1.8	3.44–13.8
LAVi (mL/m^2^)	24.7 ± 5.6	11–38.6
GLS (%)	23.2 ± 2.0	20–30
GWI (mmHg %)	2281 ± 350	1529–3518
GCW (mmHg %)	2566 ± 348	1752–3753
GWW (mmHg %)	68.4 ± 32.9	16–184
GWE (%)	96.7 ± 1.5	87–99

BP = Blood pressure, BMI = Body mass index, DT = Deceleration time, GCW = Global constructive work, GLS = Global longitudinal strain, GWE = Global work efficiency, GWI = Global work index, GWW = Global wasted work, HR = Heart rate, LAVi = Left atrial volume index, LV EF = Left ventricular ejection fraction, RDWT = Relative diastolic wall thickness.

**Table 2:** Myocardial work components according to age tertiles.

Analysis According to Age Tertiles				
Parameter	1st Tertile (<32 Years) n = 54	2d Tertile (32–<49 Years) n = 61	3rd Tertile (≥49 Years) n = 62	Cumulative *p*
GLS (%)	23.4 ± 1.9	22.8 ± 1.7	23.4 ± 2.3	0.151
GWI (mmHg %)	2230.3 ± 358.2	2288.0 ± 303.7	2319.4 ± 383.0	0.388
GCW (mmHg %)	2492.7 ± 361.9	2554.7 ± 297.0	2640.5 ± 373.3 *	<0.01
GWW (mmHg %)	70.6 ± 37.4	63.0 ± 26.2	71.7 ± 34.4	0.288
GWE (%)	96.6 ± 1.9	96.9 ± 1.1	96.6 ± 1.5	0.289
**Analysis According to HR Tertiles**				
**Parameter**	**1st Tertile** **(<66 bpm) n = 56**	**2d Tertile** **(66–<74 bpm) n = 57**	**3rd Tertile** **(≥74 bpm) n = 64**	**Cumulative *p***
GLS (%)	23.3 ± 2.0	22.8 ± 1.9	23.4 ± 2.1	0.233
GWI (mmHg %)	2294.8 ± 391.9	2272.5 ± 309.2	2277.6 ± 349.6	0.940
GCW (mmHg %)	2565.4 ± 410.1	2542.6 ± 298.4	2587.1 ± 335.3	0.783
GWW (mmHg %)	61.0 ± 32.5	67.9 ± 30.0	74.7 ± 33.6 **	<0.02
GWE (%)	96.8 ± 1.4	96.7 ± 1.2	96.5 ± 1.8	0.418

Mean values ± standard deviation with ANOVA test analysis for intergroup mean comparison; * *p* = 0.03 vs. 1st tertile; ** *p* < 0.01 vs. 1st tertile. See Table 1 for abbreviations.

**Table 3:** Independent determinants of MW components by multiple linear regression analyses.

Dependent Variable	Covariate	Β Coefficient	*p*
GWI (mmHg %)	Age (years)	−0.148	<0.001
	Systolic BP (mmHg)	0.685	<0.0001
	HR (bpm)	−0.066	<0.05
	GLS (%)	0.591	<0.0001
	E/e’ ratio	0.106	<0.02
	LAVi (mL/m^2^)	0.017	0.644
GCW (mmHg %)	Age (years)	−0.029	0.427
	Systolic BP (mmHg)	0.748	<0.0001
	HR (bpm)	−0.004	0.989
	GLS (%)	0.592	<0.0001
	E/e’ ratio	0.032	0.366
	LAVi (mL/m^2^)	−0.05	0.852
GWW (mmHg %)	Age (years)	0.014	0.890
	Systolic BP (mmHg)	0.111	0.152
	HR (bpm)	0.212	0.006
	GLS (%)	−0.200	0.008
	E/e’ ratio	0.068	0.461
	LAVi (mL/m^2^)	0.057	0.451
GWE (%)	Age (years)	−0.005	0.996
	Systolic BP (mmHg)	0.091	0.235
	HR (bpm)	−0.204	0.007
	GLS (%)	0.266	<0.0001
	E/e’ ratio	−0.117	0.206
	LAVi (mL/m^2^)	−0.078	0.302

## Supplementary Materials

The following supporting information can be downloaded at: https://www.mdpi.com/article/10.3390/diagnostics12071697/s1, Table S1. Univariable analysis for Myocardial work parameters. Table S2. Demographic and echocardiographic parameters distribution according age tertiles.
