# Supplementary material for: Impact of Age and Heart Rate on Strain-Derived Myocardial Work in a Population of Healthy Subjects
Source: Diagnostics (Basel). 2022 Jul 12;12(7):1697. doi: 10.3390/diagnostics12071697 (PMC9325020; doi:10.3390/diagnostics12071697)
Supplement: Supplementary file 1 [file diagnostics-12-01697-s001.zip › diagnostics-1792138-supplementary.pdf]

Table S1. Univariable analysis for Myocardial work parameters

Dependent variable	*Covariate*	*B coefficient*	*p*
GWI (mmHg %)	*Age (years)*	*0.121*	*0.11*
	Systolic BP (mmHg)	*0.65*	*<0.0001*
	Diastolic BP (mmHg)	*0.33*	*<0.0001*
	HR (bpm)	*-0.03*	*0.70*
	EF (%)	*0.10*	*0.17*
	E/e′ ratio	*0.22*	*0.004*
	LAVi (ml/m2)	*0.22*	*0.003*
GCW (mmHg %)	*Age (years)*	*0.19*	*0.009*
	Systolic BP (mmHg)	*0.71*	*<0.0001*
	Diastolic BP (mmHg)	*0.33*	*<0.0001*
	HR (bpm)	*-0.03*	*0.70*
	EF (%)	*0.13*	*0.08*
	E/e′ ratio	*0.21*	*0.005*
	LAVi (ml/m2)	*0.21*	*0.005*
GWW (mmHg %)	*Age (years)*	*0.02*	*0.74*
	Systolic BP (mmHg)	*0.14*	*0.06*
	Diastolic BP (mmHg)	*0.37*	*0.63*
	HR (bpm)	*0.19*	*0.01*
	EF (%)	*0.11*	*0.16*
	E/e′ ratio	*0.03*	*0.72*
	LAVi (ml/m2)	*0.03*	*0.69*
GWE (%)	*Age (years)*	*-0.007*	*0.93*
	Systolic BP (mmHg)	*0.05*	*0.55*
	Diastolic BP (mmHg)	*0.03*	*0.72*
	HR (bpm)	*-0.16*	*0.03*
	EF (%)	*-0.08*	*0.26*
	E/e′ ratio	*-0.04*	*0.611*
	LAVi (ml/m2)	*0.13*	*0.08*

Table S2. Demographic and echocardiographic parameters distribution according age tertiles

Analysis according to age tertiles				
Parameter	*1st Tertile (<32 years) n=54*	*2d Tertile (32 - <49 years) n=61*	*3rd Tertile (≥49 years) n=62*	*Cumulative p*
BMI (Kg/m²)	*21.8±2.5*	*23.9±2.6**	*24.4±2.4**	*<0.0001*
Systolic BP (mmHg)	*115.2±13.1*	*122.4±10.8***	*123.4±12.4±*	*<0.001*
Diastolic BP (mmHg)	*74.3±7.4*	*74.8±8.1*	*75.9±7.9*	*0.50*
Heart rate (bpm)	*72.4±11.4*	*70.8±9.1*	*68.9±10.5*	*0.198*
LV mass index (g/m²)	*26.3±4.7*	*30.6±7.5*	*33.3±8.1*	*<0.0001*
RDWT	*0.31±0.05*	*0.33±0.05*	*0.33±0.06*	*0.09*
LV EF	*63.5±5.08*	*63.8±5.1*	*63.5±3.8*	*0.95*
E/A ratio	*1.64±0.36*	*1.40±0.29*	*0.98±0.27*	*<0.0001*
E/e′ ratio	*5.68±0.98*	*6.32±1.24*	*7.92±2.19*	*<0.0001*
LAVi (ml/m²)	*24.3±4.9*	*24.6±5.5*	*25.1±6.2*	*0.744*
GLS (%)	*23.4 ± 1.7*	*22.5±1.6*	*22.6±1.6*	*0.01*

*p<0.0001 vs 1st tertile; ** p<0.005 vs 1st tertile, ±p<0.001 vs 1st tertile
